# Temporal trends in neonatal mortality in Pernambuco

**DOI:** 10.1590/0034-7167-2023-0451

**Published:** 2024-11-22

**Authors:** Nayara Francisca Cabral de Sousa, Ana Paula Esmeraldo Lima, Vânia Pinheiro Ramos, Mônica de Avelar Figueiredo Mafra Magalhães, André Luiz Sá de Oliveira, Eliane Rolim de Holanda, Luciana Pedrosa Leal

**Affiliations:** I Universidade Federal de Pernambuco. Recife, Pernambuco, Brazil; II Fundação Oswaldo Cruz. Rio de Janeiro, Rio de Janeiro, Brazil; III Fundação Oswaldo Cruz. Recife, Pernambuco, Brazil; IV Universidade Federal da Paraíba. João Pessoa, Paraíba, Brazil

**Keywords:** Time Serie Studies, Mortality, Neonatal, Epidemiology, Health Planning, Nursing, Estudios de Series Temporales, Mortalidade Neonatal, Epidemiología, Planificación em Salud, Enfermería

## Abstract

**Objective:**

To verify the temporal trend of neonatal mortality in the health regions of Pernambuco between 2000 and 2020.

**Method:**

A time-series ecological study was conducted, analyzing the total neonatal mortality rate and its early and late components. For regression analysis, Joinpoint Regression was applied, trends were classified, and annual and average percentage changes were calculated for the period, with a significance level of 95%.

**Results:**

The average neonatal mortality rate in Pernambuco was 11.5 during the studied period. A decreasing trend in neonatal mortality rate was observed, especially in the early component. The region where the state capital is located showed the fastest decrease across all components.

**Conclusion:**

The temporal trend of neonatal mortality was decreasing; however, the rate of reduction was not uniform across the health regions of the state, and the implementation of the Mãe Coruja Pernambucana Program did not impact the trend in neonatal mortality.

## RESUMEN

**Objetivo:**

Verificar la tendencia temporal de la mortalidad neonatal en las regiones de salud de Pernambuco entre 2000 y 2020.

**Método:**

Estudio ecológico de series temporales, donde se analizaron el coeficiente de mortalidad neonatal total y sus componentes precoz y tardío. Para el análisis de regresión, se aplicó la Regresión Joinpoint, se clasificaron las tendencias y se calcularon los porcentajes de variación anual y media en el período, con una significancia del 95%.

**Resultados:**

La tasa media de mortalidad neonatal en Pernambuco fue de 11,5 durante el período estudiado. Se observó una tendencia decreciente en la tasa de mortalidad neonatal, especialmente en el componente precoz. La región donde se encuentra la capital del estado mostró la mayor velocidad de descenso para todos los componentes.

**Conclusión:**

La tendencia temporal de la mortalidad neonatal fue decreciente; su velocidad de reducción de muertes no fue homogénea entre las regiones de salud del estado, y la implementación del Programa Mãe Coruja Pernambucana no impactó en la tendencia de la mortalidad neonatal.

## INTRODUCTION

Neonatal mortality is a principal component of infant mortality ^(1-2)^. Defined as death within the first 28 full days of life, it can be subdivided into early, occurring between zero and six days of life, or late, occurring from the seventh to the 28th day of life. From 1990 to 2019, smaller reductions in the neonatal mortality rate were observed in North America and Europe ^(3)^. In contrast, Latin America and the Caribbean, South and Southeast Asia, and Sub-Saharan Africa-regions with high neonatal mortality rates-experienced greater reductions during the same period. In Brazil, the neonatal mortality rate decreased more slowly than in other regions of the world ^(4)^, dropping from 25.33 per 1,000 live births in 1990 to 8.5 per 1,000 live births in 2019 ^(2)^.

Unlike infant mortality, neonatal mortality is not evenly distributed across all Brazilian states ^(4)^. Between 2007 and 2017, the country recorded a neonatal death rate of 9.46 per 1,000 live births ^(5)^. The highest average neonatal mortality rate in the country was in the Northern Region, at 11.02 per 1,000 live births, followed by the Northeast Region, at 10.97 per 1,000. The lowest rates were observed in the South (7.81 per 1,000) and Southeast (8.50 per 1,000) regions ^(5)^. Among the states of the Northeast Region, from 2008 to 2017, Pernambuco showed a decline in neonatal mortality greater than the national rate, predominantly in the early component ^(6-7)^.

Understanding this issue’s behavior in a territory supports health planning actions and aids in achieving Goal 3.2 of the Sustainable Development Goals (SDGs), which aims to reduce neonatal mortality in municipalities with high rates of this issue ^(8)^. The use of health information by professionals, particularly nurses due to their proximity to the enrolled population, enables organizations to build epidemiological profiles directed at planning actions to reduce neonatal death in each community ^(9)^. Identifying locations with a higher occurrence of a specific issue supports decision-making, policy planning, action implementation, and the allocation of resources to priority areas ^(10)^.

The context of high neonatal mortality rates in Pernambuco and its municipalities underscores the need to investigate contributing factors within the territory to devise strategies tailored to the needs of each health region. Greater visibility of results supports the planning and implementation of health actions with optimized resource allocation.

## OBJECTIVE

To examine the temporal trend of neonatal mortality in the health regions of Pernambuco between 2000 and 2020.

## METHODS

### Ethical Aspects

Consent from participants was waived due to the use of secondary data, and authorization for data use was requested. This study followed the Resolution No. 510 of 2016 from the National Health Council and was approved by the Ethics Committee of the Federal University of Pernambuco, under opinion number 5,509,689.

### Design, Period, and Location of the Study

**Figure 1:**
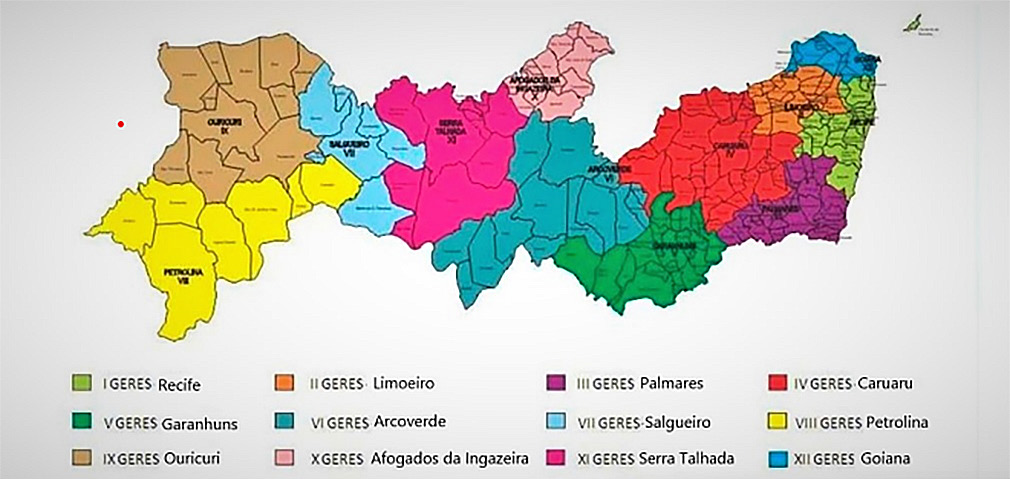
Distribution of Health Regions in Pernambuco, Brazil, 2011 ^(11)^

A time-series ecological study on neonatal mortality in the state of Pernambuco, covering the period from 2000 to 2020. The method was developed following the recommendations of the Strengthening the Reporting of Observational Studies in Epidemiology (STROBE). The State of Pernambuco, located in the Northeast region of Brazil, consists of 184 municipalities distributed across 12 Health Regions (Geres) as depicted in Figure 1: I - Recife, II - Limoeiro, III - Palmares, IV - Caruaru, V - Garanhuns, VI - Arcoverde, VII - Salgueiro, VIII - Petrolina, IX - Ouricuri, X - Afogados da Ingazeira, XI - Serra Talhada, and XII - Goiana.

### Population or Sample; Inclusion and Exclusion Criteria

The data analyzed were neonatal deaths that occurred in the health regions of Pernambuco, recorded in the Mortality Information System (SIM) and live births from the Live Birth Information System (SINASC), available on the online platform of the Department of Informatics of the Unified Health System (DATASUS). The sample included all deaths of neonates within the first 27 complete days of life.

### Study Protocol

The neonatal mortality rate was analyzed by its components: early (0 to 6 days), late (7 to 27 days), and total (0 to 27 days). For the calculation of the crude neonatal mortality rate, the number of deaths of residents aged 0 to 27 days was used as the numerator, and the total number of live births from resident mothers as the denominator, multiplied by 1000. The rates for early and late neonatal mortality were calculated using the same method, but with numerators composed of the number of live births from 0 to 6 days and from 7 to 27 days of complete life, respectively.

### Results analysis and statistics

For the analysis of the temporal trend, the Joinpoint Regression Analysis model was used. This method allows for the identification of trends in the indicators (stationary, decreasing, or increasing), their points of trend change, the annual percentage change (APC), and the average annual percent change (AAPC) over the studied period ^(12)^. In the APC and AAPC analysis, the 95% confidence interval (CI) and significance levels of 5% were considered.

The year of neonatal death was considered as the dependent variable and the number of recorded deaths as the independent variable. The Breusch-Pagan test was used to test for homoscedasticity of the residuals. The assumption of normality of the residuals was verified by the Jarque-Bera test. Temporal analysis was conducted using R software v4.1.3 and Joinpoint v4.9.1.0^(13)^, all with a significance level of 5%.

## RESULTS

In Pernambuco, 34,863 neonatal deaths were recorded from 2000 to 2020, with an average total neonatal mortality rate of 11.5. Average rates of 9.08 for early neonatal mortality and 2.42 for late neonatal mortality were also noted. The results of the temporal analysis of the rates of early, late, and total mortality are presented in Tables 1 to 3.

**Table 1:** Joinpoint regression analysis of early neonatal mortality rates, by Geres in Pernambuco for the period 2000-2020, Recife, Pernambuco, Brazil, 2023

Coefficient of early neonatal mortality	Trend 1			Trend 2			Trend 3			AAPC	95%CI
	Period	APC	95%CI	Period	APC	95%CI	Period	APC	95%CI		
Geres											
I	2000-07	-7.2^^*^^	[-9.5; -4.9]	2007-20	-2.1^^*^^	[-3.1; -1.2]				-3.9^^*^^	[-4.9; -3.0]
II	2000-20	-3.1^^*^^	[-3.9; -2.2]							-3.1^^*^^	[-3.9; -2.2]
III	2000-14	-5.9^^*^^	[-7.1; -4.7]	2014-18	7.3	[-6.4; 23.2]	2018-20	-20.0	[-39.2; 5.3]	-5.0^^*^^	[-8.3; -1.5]
IV	2000-07	-7.1^^*^^	[-9.5; -4.7]	2007-20	-1.9^^*^^	[-2.9; -0.9]				-3.8^^*^^	[-4.8; -2.8]
V	2000-03	33.0^^*^^	[0.6; 75.9]	2003-20	-3.5^^*^^	[-5.3; -1.5]				1.3	[-2.8; 5.6]
VI	2000-20	-2.1^^*^^	[3.0; -1.2]							-2.1^^*^^	[3.0; -1.2]
VII	2000-02	43.8	[-3.5; 114.3]	2002-20	-3.2^^*^^	[-4.5; -2.0]				0.7	[-3.1; 4.6]
VIII	2000-17	-2.3^^*^^	[-3.8; -0.7]	2017-20	-18.8	[-35.2; 1.8]				-5.0^^*^^	[-8.1; -1.7]
IX	2000-04	5.2	[-6.4; 18.2]	2004-10	-9.9^^*^^	[-17.0; -2.2]	2010-20	-0.3	[-3.1; 2.6]	-2.2	[-5.4; 1.1]
X	2000-20	-4.7^^*^^	[-6.5; -2.9]							-4.7^^*^^	[-6.5; -2.9]
XI	2000-04	7.3	[-4.2; 20.2]	2004-20	-4.8^^*^^	[-6.1; -3.5]				-2.5^^*^^	[-4.8; -0.2]
XII	2000-20	-3.8^^*^^	[-5.1; -2.6]							-3.8^^*^^	[-5.1; -2.6]
Pernambuco	2000-13	-4.1^^*^^	[-4.6; -3.6]	2013-20	-1.5^^*^^	[-2.9; 0.0]				-3.2^^*^^	[-3.8; -2.6]

* *p-value < 0.05; APC - Annual Percent Change; AAPC - Average Annual Percent Change over the studied period.*

**Table 2:** Joinpoint regression of late neonatal mortality rates by Geres in Pernambuco for the period 2000-2020, Recife, Pernambuco, Brazil, 2023

Coefficient of early neonatal mortality	Trend 1			Trend 2			AAPC	95%CI
	Period	APC	95%CI	Period	APC	95%CI		
Geres								
I	2000-20	-0.8^^*^^	[-1.4; -0.1]				-0.8^^*^^	[-1.4; -0.1]
II	2000-20	-0.4	[-1.6; 0.9]				-0.4	[-1.6; 0.9]
III	2000-20	-0.8	[-2.7; 1.1]				-0.8	[-2.7; 1.1]
IV	2000-20	-2.3^^*^^	[-3.6; -0.9]				-2.3^^*^^	[-3.6; -0.9]
V	2000-04	31.5^^*^^	[5.3; 64.2]	2004-20	0.3	[-2.4; 3.0]	5.8^^*^^	[1.1; 10.8]
VI	2000-20	0.8	[-1.2; 2.9]				0.8	[-1.2; 2.9]
VII	2000-20	1.3	[-1.6; 4.2]				1.3	[-1.6; 4.2]
VIII	2000-20	-2.9^^*^^	[-5.7; 0.0]				-2.9^^*^^	[-5.7; 0.0]
IX	2000-20	-1.5	[-3.3; 0.2]				-1.5	[-3.3; 0.2]
X	2000-20	-1.8	[-5.1; 1.7]				-1.8	[-5.1; 1.7]
XI	2000-20	-1.1	[-5.5; 3.5]				-1.1	[-5.5; 3.5]
XII	2000-20	-4.2^^*^^	[-7.7; -0.6]				-4.2^^*^^	[-7.7; -0.6]
Pernambuco	2000-20	-0.8^^*^^	[-1.5; -0.2]				-0.8^^*^^	[-1.5; -0.2]

* *p-value < 0.05; APC - Annual Percent Change; AAPC - Average Annual Percent Change over the studied period.*

The rates of early neonatal mortality displayed statistically significant trends across all Geres (Table 1). Geres II, VI, X, and XII exhibited a uniform decline in trends from 2000 to 2020. Geres I, III, IV, V, VII, VIII, IX, and XI experienced changes in the pattern of trend behavior for early neonatal mortality.

Geres I showed a more pronounced declining trend (APC = -7.2) from 2000 to 2007 and a more attenuated trend from 2008 (APC = -2.1), with an AAPC of -3.9. Geres II also displayed a declining trend, with a significant AAPC of -3.1 for the entire period.

Geres III recorded two changes in the behavior of the trend of early neonatal mortality. From 2000 to 2014, the trend of the variable was decreasing with an APC of -5.9. From 2015 to 2018, there was a change in behavior when it became ascending with an APC of 7.3. The last change in the trend occurred between 2019 and 2020, during which the behavior returned to decreasing, but more pronounced than initially, with an APC of -20.0. Overall, Geres III exhibited a decreasing trend behavior, with an AAPC of -5.0.

Geres IX exhibited modifications in the behavior of the trends of early neonatal mortality. From 2000 to 2004, it was ascending, with an APC of 5.2. This was followed by a marked decline from 2004 to 2010 (APC of -9.9) and a period from 2011 to 2020, during which the behavior of the variable continued to decrease, albeit more discreetly (APC of -0.3). Thus, Geres IX showed a decline in early neonatal death rates with an AAPC of -2.2.

In Pernambuco, the rate of early neonatal mortality exhibited a significant declining trend, more pronounced from 2000 to 2013 and more subtle starting in 2014, with an AAPC of -3.2.

The late mortality rates (Table 2) showed divergences among trends in Geres I, IV, V, VIII, and XII. From 2000 to 2020, there was a trend of reduction in late neonatal mortality rates in Geres I, IV, VIII, and XII, and an increase in Geres V from 2000 to 2004. Geres V exhibited an inflection point in the growth of the late neonatal mortality rate between 2005 and 2020, although without statistical significance. Thus, in Pernambuco, a temporal trend of reduction was observed throughout the period, with an AAPC of -0.8.

**Table 3:** Joinpoint regression of total neonatal mortality rates by Geres in Pernambuco for the period 2000-2020, Recife, Pernambuco, Brazil, 2023

Coefficient of early neonatal mortality	Trend 1			Trend 2			Trend 3			AAPC	95%CI
	Period	APC	95%CI	Period	APC	95%CI	Period	APC	95%CI		
Geres											
I	2000-04	-9.4^^*^^	[-13.6; -5.0]	2004-20	-2.2^^*^^	[-2.7; -1.6]				-3.7^^*^^	[-4.6; -2.7]
II	2000-20	-2.6^^*^^	[-3.3; -1.8]							-2.6^^*^^	[-3.3; -1.8]
III	2000-14	-5.1^^*^^	[-6.2; -4.0]	2014-18	6.5	[-5.8; 20.3]	2018-20	-17.4	[-35.3;5.4]	-4.2^^*^^	[-7.3; -1.1]
IV	2000-08	-5.6^^*^^	[-7.6; -3.6]	2008-20	-1.9^^*^^	[-3.0; -0.7]				-3.4^^*^^	[-4.3; -2.4]
V	2000-03	33.8^^*^^	[6.1; 68.8]	2003-20	-2.8^^*^^	[-4.4; -1.3]				1.9	[-1.5; 5.5]
VI	2000-20	-1.7^^*^^	[-2.6; -0.8]							-1.7^^*^^	[-2.6; -0.8]
VII	2000-02	44.9^^*^^	[6.9; 96.4]	2002-20	-2.8^^*^^	[-3.7; -1.8]				1.2	[-1.7; 4.2]
VIII	2000-16	-2.0^^*^^	[-3.5; -0.4]	2016-20	-14.7^^*^^	[-25.1; -2.9]				-4.7^^*^^	[-7.2; -2.1]
IX	2000-04	5.5	[-5.5; 17.8]	2004-10	-9.0^^*^^	[-15.8; -1.6]	2010-20	-0.4	[-3.0; 2.4]	-1.9	[-5.0; 1.2]
X	2000-20	-4.1^^*^^	[-5.6; -2.7]							-4.1^^*^^	[-5.6; -2.7]
XI	2000-04	7.6	[-4.2; 21.0]	2004-20	-4.4^^*^^	[-5.8; -3.1]				-2.1	[-4.4; 0.3]
XII	2000-20	-3.7^^*^^	[-5.1; -2.4]							-3.7^^*^^	[-5.1; -2.4]
Pernambuco	2000-20	-2.9^^*^^	[-3.2; -2.5]							-2.9^^*^^	[-3.2; -2.5]

* *p-value < 0.05; APC - Annual Percent Change; AAPC - Average Annual Percent Change over the studied period.*

The total neonatal mortality rate (Table 3) showed decreasing trends for all Geres, except for Geres IX and XI. Geres II, VI, X, and XII exhibited uniform behavior throughout the studied period. Inflection points, with changes in behavior or smoothing of the trend in total neonatal mortality, were observed in Geres I, III, IV, V, VII, VIII, IX, and XI.

Geres I demonstrated a decreasing trend throughout the entire study period, although there was a smoothing in the reduction between 2005 and 2020 (APC of -2.2). In Geres III, between 2000 and 2014, there was a decreasing trend (APC of -5.1), followed by growth between 2015 and 2018 (APC of 6.5) and a change in trend behavior with a new decrease between 2018 and 2020 (APC of -17.4).

Geres V and VII registered a marked upward pattern in the first period of the study for mortality rates and a downward pattern in the second period. Although not statistically significant, Geres IX exhibited two inflection points: between 2000 and 2004, the behavior was ascending (APC of 5.5), followed by a descending interval between 2005 and 2010 (APC of -9.0), and in the last interval from 2011 to 2020, the trend continued to decline (APC of -0.4), albeit at a slower pace.

Overall, the state of Pernambuco showed a pattern of decline in neonatal mortality rates between the years 2000 and 2019, with an AAPC of -2.9.

## DISCUSSION

In this study, a reduction in the mortality rate in Pernambuco between 2000 and 2020 was observed, with divergent patterns among the health regions. Local disparities may vary according to the level of social and economic development in each region, affecting the structure and quality of maternal and infant care services, and impacting neonatal mortality indicators ^(14)^.

The annual rate of reduction in the total neonatal mortality rate in the State of Pernambuco was 2.9% between 2000 and 2020. This reduction has also been observed in other studies ^(5)^. In Brazil, neonatal mortality is considered preventable through timely and effective actions by health services ^(10)^. The Northeast region exhibited the highest rates of reduction in infant mortality coefficients, as a result of expanded access to primary care actions and improvements in health service infrastructure, directly reducing regional disparities related to deaths in this age group ^(3,15)^.

Regional differences associated with mortality in the first years of life can be exacerbated by local socioeconomic conditions ^(16)^. As a result, the spatial distribution of neonatal mortality does not occur randomly across various regions of the world, and its coefficients decrease more quickly in developed countries ^(4)^. Rates vary within the same territory, correlating the role of geographical spaces and their borders with populations at risk for certain health issues ^(17)^.

The decrease in Brazilian infant mortality rates is associated with universal access to health services provided by the Family Health Strategy, focusing on actions that promote and prevent child health issues ^(18)^. In this context, ongoing health programs in the state, such as the Mãe Coruja Pernambucana, aim to provide comprehensive care to pregnant women using the Unified Health System (SUS) and to their babies.

The behavior of total neonatal mortality in Geres V, VII, IX, and XI, starting in 2000, was increasing until 2004 and, in subsequent years, followed the decreasing trend observed in other regions of the state until 2020. In these regions, the Mãe Coruja Pernambucana Program (PMCPE) was implemented in 2007 (IX), 2009 (V and XI), and 2012 (VII), reaffirming that the implementation of this program did not correlate with changes in mortality behavior in the participating municipalities.

The profile of the decline in neonatal mortality rates in Pernambuco is similar to that of Brazil ^(6)^. Geres II, VI, X, and XII exhibited a uniform and decreasing trend throughout the analyzed period. The implementation of the PMCPE in the municipalities of these regions occurred in 2010, without affecting the decreasing behavior of these Geres.

In the state, the PMCPE was initiated in 2007 with the aim of improving maternal and infant mortality rates through enhanced prenatal care, childbirth services, and newborn care ^(14)^. However, no changes or accentuations in trends among the studied variables were observed following its implementation, a result similar to another study conducted in this state ^(7)^.

The lack of prioritization of the PMCPE on interventions that have a greater impact on neonatal mortality, along with its implementation at different times in some Geres, may have contributed to the absence of significant results on the coefficients of neonatal mortality rates during the analyzed period ^(7)^. Additionally, the random implementation of the program without systematic territorial continuity and with a focus exclusively on regions with the highest neonatal death rates may also have influenced the absence of positive results of the program on the variables related to neonatal death.

Among the components of the neonatal mortality rate, the rate of decline of the early component was approximately four times greater than that of the late component in the state. Geres V, VII, IX, and XI exhibited an increasing trend for early neonatal mortality from 2000 to 2004, followed by a decreasing trend from that period until the end of the temporal cut-off. The initial increase in the series for the early component occurred due to the absence of specialized services (Geres V), the territorial distance from equipped centers providing this type of assistance (VII and XI), and a pre-existing high mortality rate already recorded in other studies ^(6,19)^. In Pernambuco, neonatal mortality evolved with sustained reductions in both early and late components, with the rate of decline of the early component being higher than that of the total, confirming the trend presented in other studies ^(3-7)^.

Late neonatal mortality exhibited a uniform and decreasing behavior in all Geres, except in Geres V, VI, and VII. However, only Geres V showed statistical significance for this component. Late neonatal death is associated with preventable and reducible causes through qualified attention to women during pregnancy, childbirth, and to the newborn ^(17)^. In Geres V, the main cause of hospitalization in women of childbearing age is linked to illnesses in the pregnancy-puerperal cycle, confirming the results obtained in this study ^(20)^.

### Study Limitations

This study was limited by the use of secondary data, which depend on the completeness of information in the data systems fed by records generated by health services. However, studies utilizing information systems offer benefits such as low cost and speed and also allow for a broader observation of a particular health issue, with the development of indicators that support the planning and implementation of health interventions.

### Contributions to Nursing, Health, or Public Policy

The findings of this study broaden the understanding of neonatal death in Pernambuco and highlight the need for new strategies aimed at reducing this issue, targeted at areas with the highest concentration of notifications. Nurses, as health managers, can use this information to plan health promotion actions aimed at reducing neonatal mortality, grounded in situational reality, and consequently, help close the gap in achieving SDG 3.2.

## CONCLUSIONS

The trend of neonatal mortality and its components in the state of Pernambuco was decreasing. However, the early component exhibited a rate of reduction four times greater than the late component. It is notable that despite the reduction in coefficients, the rate of reduction in neonatal deaths is not uniform across all regions of the state.

In Pernambuco, even after the implementation of the PMCPE, no significant results were identified concerning the trend in neonatal mortality. The development of systematic and continuous actions from prenatal care to birth across the entire territory of Pernambuco could contribute to a significant reduction in the state’s neonatal mortality rates, addressing the main variables that influence neonatal death.

Thus, it was observed that conducting studies with secondary data enables the identification of critical and transitional areas that most require intervention. Another benefit of this type of study is that the information obtained supports the development of a strategic plan tailored to local realities, aimed at reducing neonatal mortality rates.

## References

[1] Lansky S, Friche AAL, Silva AAM, Campos D, Bittencourt SDA, Carvalho ML. (2014). Pesquisa nascer no Brasil: perfil da mortalidade neonatal e avaliação da assistência à gestante e ao recém-nascido. Cad Saúde Pública.

[2] World Health Organization (WHO) (2019). Levels & trends in child mortality report 2019: estimates developed by the UM Inter-agency Group for Child Mortality Estimation.

[3] Szwarcwald CL, Almeida WS, Teixeira RA, França EB, Miranda MJ, Malta DC. (2020). Inequalities in infant mortality in Brazil at subnational levels in Brazil, 1990 to 2015. Popul Health Metr.

[4] Wang S, Ren Z, Liu X. (2023). Spatio temporal trends in neonatal, infant, and child mortality (1990-2019) based on Bayesian spatio temporal modeling. Front Public Health.

[5] Bernardino FBS, Gonçalves TM, Pereira TID, Xavier JS, Freitas BHBM, Gaiva MAM. (2022). Tendência da mortalidade neonatal no Brasil de 2007 a 2017. Ciênc saúde colet (Online).

[6] Gomes AGN, Freitas SMS, Pontestma A, Portela NM, Pimentel FC. (2022). Mortalidade neonatal em Pernambuco: tendência, distribuição geográfica e perfil dos óbitos de 2008 a 2017. Rev Pesqui Cuid Fundam.

[7] Instituto de Pesquisa Econômica Aplicada (IPEA) (2018). Agenda 2030: ODS - Metas Nacionais dos Objetivos de Desenvolvimento Sustentável.

[8] Ferreira AG, Carvalho DP, Barlem ELD, Rocha LP, da Silva MRS, Vaz MRC. (2019). Participação Social na Saúde e o Papel da Enfermagem: aplicação do modelo ecológico. Rev Pesqui Cuid Fundam.

[9] Bonifácio SR, Lopes EL. (2019). Mapeamento de agravos de saúde: uma aplicação da técnica de georreferenciamento com o uso do software Google Earth. Int J Health Manag Rev.

[10] Lima SS, Braga MC, Vanderlei LCM, Luna CF, Frias PG. (2020). Avaliação do impacto de programas de assistência pré-natal, parto e ao recém-nascido nas mortes evitáveis em Pernambuco, Brasil: estudo de adequação. Cad Saúde Pública.

[11] Secretaria Estadual de Saúde de Pernambuco (2011). Plano Diretor de Regionalização: Recife.

[12] Souza CDF, Luna CF, Magalhães MAFM. (2019). Transmissão da hanseníase na Bahia, 2001-2015: modelagem a partir da regressão por pontos de inflexão e estatística de varredura espacial. Epidemiol Serv Saúde.

[13] National Cancer Institute (US), Surveillance Research Program (2018). Joinpoint Regression Program version 4.6.: Statistical Methodology and Applications Branch.

[14] Prezotto KH, Oliveira RR, Pelloso SM, Fernandes CAM. (2021). Tendência da mortalidade neonatal evitável nos Estados do Brasil. Rev Bras Saúde Matern Infant.

[15] Souza CDF, Albuquerque AR, Cunha EJO, Silva LCF, Silva JVM, Santos FGB (2021). Novo século, velho problema: tendência da mortalidade infantil e seus componentes no Nordeste brasileiro. Cad Saúde Colet.

[16] Amegbor PM, Addae A. (2023). Spatiotemporal analysis of the effect of global development indicators on child mortality. Int J Health Geogr.

[17] Burstein R, Henry NJ, Collison ML, Marczak LB, Sligar A, Watson S (2019). Mapping 123 million neonatal, infant and child deaths between 2000 and 2017. Nature.

[18] Adamski K, Silva TG, Pereira PPS, Farias ES, Cantarelli KJ, Mendes VA. (2022). Mortalidade por causas evitáveis em macrorregiões de saúde: série temporal 2007 a 2020. REAS.

[19] Secretaria Estadual de Saúde de Pernambuco, V Gerência Regional de Saúde (2021). Mapa da Saúde da V Região de Saúde.

[20] Baptista GC, Poton WL. (2021). Evolução da mortalidade neonatal por causas evitáveis no Espírito Santo ao longo de dez anos. Rev Bras Saúde Matern Infant.

