# Supplementary material for: Temporal trends in neonatal mortality in Pernambuco
Source: Rev Bras Enferm. 2024 Nov 22;77(4):e20230451. doi: 10.1590/0034-7167-2023-0451 (PMC11654227; doi:10.1590/0034-7167-2023-0451)
Supplement: Translation

# Translation: Tendência temporal da mortalidade neonatal em Pernambuco

**DOI:** 10.1590/0034-7167-2023-0451pt

**Authors:** Nayara Francisca Cabral de Sousa, Ana Paula Esmeraldo Lima, Vânia Pinheiro Ramos, Mônica de Avelar Figueiredo Mafra Magalhães, André Luiz Sá de Oliveira, Eliane Rolim de Holanda, Luciana Pedrosa Leal

**Affiliations:** I Universidade Federal de Pernambuco. Recife, Pernambuco, Brasil; II Fundação Oswaldo Cruz. Rio de Janeiro, Rio de Janeiro, Brasil; III Fundação Oswaldo Cruz. Recife, Pernambuco, Brasil; IV Universidade Federal da Paraíba. João Pessoa, Paraíba, Brasil

**Keywords:** Estudos de Séries Temporais, Mortalidade Neonatal, Epidemiologia, Planejamento em Saúde, Enfermagem

## Abstract

**Objetivo:**

Verificar a tendência temporal da mortalidade neonatal nas regionais de saúde de Pernambuco entre 2000 e 2020.

**Método:**

Estudo ecológico de série temporal, onde foram analisados o coeficiente de mortalidade neonatal total e seus componentes precoce e tardio. Para análise de regressão, foi aplicado o *Joinpoint Regression*, classificadas as tendências e calculados percentuais de variação anual e média no período, com significância de 95%.

**Resultados:**

A taxa média de mortalidade neonatal em Pernambuco foi 11,5 no período estudado. Foi observada tendência decrescente da taxa de óbito neonatal, especialmente no componente precoce. A regional onde se localiza a capital do estado apresentou maior velocidade de queda para todos os componentes.

**Conclusão:**

A tendência temporal da mortalidade neonatal foi decrescente; sua velocidade de redução dos óbitos não foi homogênea entre as regiões de saúde do estado, e a implantação do Programa Mãe Coruja Pernambucana não impactou na tendência da mortalidade neonatal.

## INTRODUÇÃO

O óbito neonatal é o principal componente da mortalidade infantil^(1,2)^. Definido como o óbito nos primeiros 28 dias completos de vida, pode ser subdividido em precoce, quando ocorre entre zero e seis dias de vida, ou tardio, quando acontece do sétimo ao 28º dia de vida. No período de 1990 a 2019, em países da América do Norte e Europa, foram evidenciadas menores reduções na razão de mortalidade neonatal^(3)^. A América Latina e Caribe, Sul e Sudeste da Ásia e África Subsaariana, regiões com taxas de mortalidade neonatal elevadas, apresentaram maiores reduções no mesmo período. No Brasil, a taxa de mortalidade neonatal reduziu mais lentamente que em outras regiões do mundo^(4)^, passando de 25,33/1.000 nascidos vivos (NV) em 1990 para 8,5/1.000 NV em 2019^(2)^.

Ao contrário do que ocorre na mortalidade infantil, a mortalidade neonatal não se distribui de forma homogênea em todos os estados brasileiros ^(4)^. O país apresentou, entre 2007 e 2017, uma taxa de óbito neonatal de 9,46/1.000 nascidos vivos ^(5)^. A maior taxa média de mortalidade neonatal no país foi na Região Norte, com 11,02/1.000 nascidos vivos, seguida pela Região Nordeste, com 10,97/1.000. As menores taxas estão nas regiões Sul (7,81/1.000) e Sudeste (8,50/1.000) ^(5)^. Entre os estados da Região Nordeste, no período de 2008 a 2017, Pernambuco apresentou uma queda da mortalidade neonatal superior à taxa nacional, com predominância no componente precoce ^(6,7)^.

O conhecimento do comportamento desse agravo em um território fornece suporte para ações de planejamento em saúde e auxilia no cumprimento da meta 3.2 dos Objetivos de Desenvolvimento Sustentável (ODS), que visa à redução da mortalidade neonatal nos municípios com elevadas taxas desse agravo ^(8)^. O uso da informação em saúde por profissionais e, mais especificamente, pelo enfermeiro, devido à sua proximidade com a população adscrita, possibilita a organização para a construção de perfis epidemiológicos direcionados ao planejamento de ações para redução do óbito neonatal em cada comunidade ^(9)^. A identificação de locais com maior ocorrência de determinado agravo subsidia a tomada de decisão, o planejamento de políticas, a implantação de ações e a alocação de recursos direcionados a áreas prioritárias ^(10)^.

O contexto das elevadas taxas de mortalidade neonatal em Pernambuco e em seus municípios desperta a necessidade de investigação dos fatores contribuintes para esse agravo no território, para realizar estratégias conforme a necessidade de cada região de saúde. A maior visibilidade sobre os resultados oferece subsídios para o planejamento e implantação de ações em saúde com a otimização de recursos direcionados.

## OBJETIVO

Verificar a tendência temporal da mortalidade neonatal nas regionais de saúde de Pernambuco entre 2000 e 2020.

## MÉTODOS

### Aspectos éticos

O consentimento dos participantes da pesquisa foi dispensado devido à utilização de dados secundários, e a autorização para uso dos dados foi solicitada. O presente estudo seguiu a Resolução nº 510 de 2016 do Conselho Nacional de Saúde e foi aprovado pelo Comitê de Ética da Universidade Federal de Pernambuco.

### Desenho, período e local do estudo

Estudo ecológico de séries temporais sobre a mortalidade neonatal no estado de Pernambuco, no período de 2000 a 2020. Para elaboração do método, foram utilizadas as recomendações do *Strengthening the Reporting of Observational Studies in Epidemiology* (STROBE). O Estado de Pernambuco, situado na região Nordeste do Brasil, é composto por 184 municípios, distribuídos em 12 Regiões de Saúde (Geres), conforme a Figura 1: I - Recife, II - Limoeiro, III - Palmares, IV - Caruaru, V - Garanhuns, VI - Arcoverde, VII - Salgueiro, VIII - Petrolina, IX - Ouricuri, X - Afogados da Ingazeira, XI - Serra Talhada e XII - Goiana.

**Figura 1:** Distribuição das Regiões em Saúde de Pernambuco, Brasil, 2011^(11)^

### População ou amostra; critérios de inclusão e exclusão

Os dados analisados foram os óbitos neonatais ocorridos nas regiões de saúde de Pernambuco, registrados no Sistema de Informação sobre Mortalidade (SIM) e os nascidos vivos do Sistema de Informações sobre Nascidos Vivos (SINASC), disponibilizados pelo Ministério da Saúde na plataforma online do Departamento de Informática do Sistema Único de Saúde (DATASUS). Foram selecionados para a amostra todos os óbitos ocorridos em neonatos nos primeiros 27 dias completos de vida.

### Protocolo do estudo

A taxa de mortalidade neonatal foi analisada por seus componentes: precoce (0 a 6 dias), tardio (7 a 27 dias) e total (0 a 27 dias). Para o cálculo da taxa bruta da mortalidade neonatal, foi utilizado o número de óbitos de residentes de 0 a 27 dias como numerador, e o número total de nascidos vivos de mães residentes como denominador, multiplicado por 1000. A taxa de mortalidade neonatal precoce e tardia foi calculada pelo mesmo método, mas com os numeradores compostos pelo número de nascidos vivos de 0 a 6 dias e de 7 a 27 dias de vida completos, respectivamente.

### Análise dos resultados e estatística

Para análise da tendência temporal foi utilizado o modelo de regressão por pontos de inflexão (*Joinpoint Regression Analysis*). Por esse método é possível identificar a tendência dos indicadores (estacionária, decrescente ou crescente), seus pontos de modificação da tendencia, a variação percentual anual (APC) e ao longo do período estudado (AAPC)^(12)^. Na análise AAPC e APC foram considerados o IC95% e níveis de significância de 5%.

O ano do óbito neonatal foi considerado como variável dependente e o número de óbitos registrados como variável independente. Para testar a homoscedasticidade dos resíduos foi utilizado do teste de Breusch-Pagan. A suposição de normalidade dos resíduos foi verificada pelo teste Jarque-Bera. A análise temporal foi realizada no software R v4.1.3 e *Joinpoint* v.4.9.1.0^(13)^, todos com significância de 5%.

## RESULTADOS

Em Pernambuco, foram registrados 34.863 óbitos neonatais no período de 2000 a 2020, com uma taxa média de mortalidade neonatal total de 11,5. Para seus componentes, foram encontradas taxas médias de 9,08 para o precoce e 2,42 para o tardio. Os resultados da análise temporal das taxas de mortalidade precoce, tardia e total estão apresentados nas tabelas de 1 a 3.

As taxas de mortalidade precoce apresentaram tendências estatisticamente significantes em todas as Geres (Tabela 1). As Geres II, VI, X e XII apresentaram um declínio uniforme na tendência entre os anos de 2000 a 2020. As Geres I, III, IV, V, VII, VIII, IX e XI cursaram com mudança no padrão de comportamento da tendência de mortalidade neonatal precoce.

**Tabela 1:** Regressão por pontos de inflexão da taxa de mortalidade neonatal precoce, por Geres em Pernambuco no período de 2000-2020, Recife, Pernambuco, Brasil, 2023

Coeficiente de mortalidade neonatal precoce	Tendência 1			Tendência 2			Tendência 3			AAPC	IC 95%
	Período	APC	IC 95%	Período	APC	IC 95%	Período	APC	IC 95%		
Geres											
I	2000-07	-7,2^^*^^	[-9,5; -4,9]	2007-20	-2,1^^*^^	[-3,1; -1,2]				-3,9^^*^^	[-4,9; -3,0]
II	2000-20	-3,1^^*^^	[-3,9; -2,2]							-3,1^^*^^	[-3,9; -2,2]
III	2000-14	-5,9^^*^^	[-7,1; -4,7]	2014-18	7,3	[-6,4; 23,2]	2018-20	-20,0	[-39,2; 5,3]	-5,0^^*^^	[-8,3; -1,5]
IV	2000-07	-7,1^^*^^	[-9,5; -4,7]	2007-20	-1,9^^*^^	[-2,9; -0,9]				-3,8^^*^^	[-4,8; -2,8]
V	2000-03	33,0^^*^^	[0,6; 75,9]	2003-20	-3,5^^*^^	[-5,3; -1,5]				1,3	[-2,8; 5,6]
VI	2000-20	-2,1^^*^^	[3,0; -1,2]							-2,1^^*^^	[3,0; -1,2]
VII	2000-02	43,8	[-3,5; 114,3]	2002-20	-3,2^^*^^	[-4,5; -2,0]				0,7	[-3,1; 4,6]
VIII	2000-17	-2,3^^*^^	[-3,8; -0,7]	2017-20	-18,8	[-35,2; 1,8]				-5,0^^*^^	[-8,1; -1,7]
IX	2000-04	5,2	[-6,4; 18,2]	2004-10	-9,9^^*^^	[-17,0; -2,2]	2010-20	-0,3	[-3,1; 2,6]	-2,2	[-5,4; 1,1]
X	2000-20	-4,7^^*^^	[-6,5; -2,9]							-4,7^^*^^	[-6,5; -2,9]
XI	2000-04	7,3	[-4,2; 20,2]	2004-20	-4,8^^*^^	[-6,1; -3,5]				-2,5^^*^^	[-4,8; -0,2]
XII	2000-20	-3,8^^*^^	[-5,1; -2,6]							-3,8^^*^^	[-5,1; -2,6]
Pernambuco	2000-13	-4,1^^*^^	[-4,6; -3,6]	2013-20	-1,5^^*^^	[-2,9; 0,0]				-3,2^^*^^	[-3,8; -2,6]

^^*^^Valor de p<0,05; APC - Variação percentual anual; AAPC - Variação ao longo do período estudado.

A Geres I mostrou uma tendência mais acentuada de queda (APC = -7,2) no período de 2000 a 2007 e uma tendência mais atenuada a partir de 2008 (APC = -2,1), com AAPC de -3,9. A Geres II também apresentou tendência de queda, sendo a AAPC significante no período total de -3,1.

A Geres III registrou duas alterações no comportamento da tendência da mortalidade neonatal precoce. Entre 2000 e 2014, a tendência da variável foi decrescente com APC de -5,9, enquanto no período entre 2015 e 2018 houve uma mudança no comportamento, quando se comportou de forma ascendente com APC de 7,3; a última alteração da tendência foi registrada entre 2019 e 2020, no qual o comportamento voltou a ser decrescente, porém mais acentuado que o inicial, com APC de -20,0. De maneira geral, a Geres III apresentou tendência de comportamento decrescente, com AAPC de -5,0.

A Geres IX exibiu modificações no comportamento das tendências da mortalidade neonatal precoce. No período de 2000 a 2004 foi ascendente, com APC de 5,2, seguida pelos anos de 2004 a 2010, onde houve uma acentuada queda nas taxas de óbito neonatal precoce, (APC de -9,9) e o período entre 2011 e 2020, cujo comportamento da variável continuou decrescente, porém de forma mais discreta (APC de -0,3). Dessa forma, a Geres IX apresentou queda nas taxas de óbito neonatal precoce com AAPC de -2,2.

Em Pernambuco, a taxa de mortalidade neonatal precoce teve uma tendência de queda significativa, mais acentuada no período de 2000 a 2013 e uma tendência de queda mais tênue a partir de 2014, sendo a AAPC de -3,2.

As taxas de mortalidade tardia (Tabela 2) apresentaram divergências entre as tendências nas Geres I, IV, V, VIII e XII. No período de 2000 a 2020, houve tendência de redução das taxas de mortalidade neonatal tardia nas Geres I, IV, VIII e XII e de crescimento na Geres V, de 2000 a 2004. A Geres V apresentou um ponto de inflexão no crescimento da taxa de mortalidade neonatal tardia entre 2005 e 2020, embora sem significância estatística. Assim, em Pernambuco, observou-se a tendência temporal de redução em todo o período, com uma AAPC de -0,8.

**Tabela 2:** Regressão por pontos de inflexão da taxa de mortalidade neonatal tardia, por Geres em Pernambuco no período de 2000-2020, Recife, Pernambuco, Brasil, 2023

Coeficiente de mortalidade neonatal tardia	Tendência 1			Tendência 2			AAPC	IC 95%
	Período	APC	IC 95%	Período	APC	IC 95%		
Geres								
I	2000-20	-0,8^^*^^	[-1,4; -0,1]				-0,8^^*^^	[-1,4; -0,1]
II	2000-20	-0,4	[-1,6; 0,9]				-0,4	[-1,6; 0,9]
III	2000-20	-0,8	[-2,7; 1,1]				-0,8	[-2,7; 1,1]
IV	2000-20	-2,3^^*^^	[-3,6; -0,9]				-2,3^^*^^	[-3,6; -0,9]
V	2000-04	31,5^^*^^	[5,3; 64,2]	2004-20	0,3	[-2,4; 3,0]	5,8^^*^^	[1,1; 10,8]
VI	2000-20	0,8	[-1,2; 2,9]				0,8	[-1,2; 2,9]
VII	2000-20	1,3	[-1,6; 4,2]				1,3	[-1,6; 4,2]
VIII	2000-20	-2,9^^*^^	[-5,7; 0,0]				-2,9^^*^^	[-5,7; 0,0]
IX	2000-20	-1,5	[-3,3; 0,2]				-1,5	[-3,3; 0,2]
X	2000-20	-1,8	[-5,1; 1,7]				-1,8	[-5,1; 1,7]
XI	2000-20	-1,1	[-5,5; 3,5]				-1,1	[-5,5; 3,5]
XII	2000-20	-4,2^^*^^	[-7,7; -0,6]				-4,2^^*^^	[-7,7; -0,6]
Pernambuco	2000-20	-0,8^^*^^	[-1,5; -0,2]				-0,8^^*^^	[-1,5; -0,2]

^^*^^Valor de p<0,05; APC - Variação percentual anual; AAPC - Variação ao longo do período estudado.

A taxa de mortalidade neonatal total (Tabela 3) mostrou tendências decrescentes para todas as Geres, exceto as Geres IX e XI. As Geres II, VI, X e XII apresentaram um comportamento homogêneo no período estudado. A ocorrência de pontos de inflexão, com mudança de comportamento ou suavização da tendência de mortalidade neonatal total, foi observada nas Geres I, III, IV, V, VII, VIII, IX e XI.

**Tabela 3:** Regressão por pontos de inflexão da taxa de mortalidade neonatal total, por Geres em Pernambuco no período de 2000-2020, Recife, Pernambuco, Brasil, 2023

Coeficiente de mortalidade neonatal	Tendência 1			Tendência 2			Tendência 3			AAPC	IC 95%
	Período	APC	IC 95%	Período	APC	IC 95%	Período	APC	IC 95%		
Geres											
I	2000-04	-9,4^^*^^	[-13,6; -5,0]	2004-20	-2,2^^*^^	[-2,7; -1,6]				-3,7^^*^^	[-4,6; -2,7]
II	2000-20	-2,6^^*^^	[-3,3; -1,8]							-2,6^^*^^	[-3,3; -1,8]
III	2000-14	-5,1^^*^^	[-6,2; -4,0]	2014-18	6,5	[-5,8; 20,3]	2018-20	-17,4	[-35,3;5,4]	-4,2^^*^^	[-7,3; -1,1]
IV	2000-08	-5,6^^*^^	[-7,6; -3,6]	2008-20	-1,9^^*^^	[-3,0; -0,7]				-3,4^^*^^	[-4,3; -2,4]
V	2000-03	33,8^^*^^	[6,1; 68,8]	2003-20	-2,8^^*^^	[-4,4; -1,3]				1,9	[-1,5; 5,5]
VI	2000-20	-1,7^^*^^	[-2,6; -0,8]							-1,7^^*^^	[-2,6; -0,8]
VII	2000-02	44,9^^*^^	[6,9; 96,4]	2002-20	-2,8^^*^^	[-3,7; -1,8]				1,2	[-1,7; 4,2]
VIII	2000-16	-2,0^^*^^	[-3,5; -0,4]	2016-20	-14,7^^*^^	[-25,1; -2,9]				-4,7^^*^^	[-7,2; -2,1]
IX	2000-04	5,5	[-5,5; 17,8]	2004-10	-9,0^^*^^	[-15,8; -1,6]	2010-20	-0,4	[-3,0; 2,4]	-1,9	[-5,0; 1,2]
X	2000-20	-4,1^^*^^	[-5,6; -2,7]							-4,1^^*^^	[-5,6; -2,7]
XI	2000-04	7,6	[-4,2; 21,0]	2004-20	-4,4^^*^^	[-5,8; -3,1]				-2,1	[-4,4; 0,3]
XII	2000-20	-3,7^^*^^	[-5,1; -2,4]							-3,7^^*^^	[-5,1; -2,4]
Pernambuco	2000-20	-2,9^^*^^	[-3,2; -2,5]							-2,9^^*^^	[-3,2; -2,5]

^^*^^Valor de p<0,05; APC - Variação percentual anual; AAPC - Variação ao longo do período estudado.

A Geres I demonstrou um comportamento decrescente ao longo de todo o período de estudo, embora tenha ocorrido uma suavização na redução entre 2005 e 2020 (APC de -2,2). Na Geres III, entre os anos de 2000 e 2014, houve uma tendência decrescente (APC de -5,1), seguida por um crescimento entre 2015 e 2018 (APC de 6,5) e por uma mudança de comportamento da tendência com um novo decréscimo entre 2018 e 2020 (APC de -17,4).

As Geres V e VII registraram um acentuado padrão ascendente no primeiro período do estudo para as taxas de mortalidade e descendente no segundo período. Embora sem significância estatística, a Geres IX apresentou dois pontos de inflexão: entre 2000 e 2004, o comportamento foi ascendente (APC de 5,5), seguido por um intervalo descendente entre 2005 e 2010 (APC de -9,0), e no último intervalo de 2011 a 2020, a tendência continuou descendente (APC de -0,4), porém com menor velocidade.

No geral, o estado de Pernambuco apresentou um padrão de queda nas taxas de mortalidade neonatal entre os anos de 2000 e 2019, com uma AAPC de -2,9.

## DISCUSSÃO

Neste estudo, observou-se uma redução na taxa de mortalidade em Pernambuco entre 2000 e 2020, com padrões divergentes entre as regionais de saúde. Disparidades locais podem variar conforme o nível de desenvolvimento social e econômico de cada região, afetando a estruturação e a qualidade dos serviços de atenção materno-infantil e repercutindo sobre os indicadores de mortalidade neonatal^(14)^.

A velocidade da redução anual da taxa de mortalidade neonatal total no Estado de Pernambuco foi de 2,9% entre 2000 e 2020. Essa redução também tem sido observada em outros estudos^(5)^. No Brasil, a mortalidade neonatal é considerada evitável por meio de ações oportunas e efetivas dos serviços de saúde^(10)^. A região Nordeste apresentou as maiores taxas de redução nos coeficientes de mortalidade infantil, como consequência da expansão do acesso às ações de Atenção Primária e da melhoria na infraestrutura dos serviços de saúde, reduzindo diretamente as disparidades regionais relacionadas ao óbito nessa faixa etária^(3,15)^.

As diferenças regionais associadas à mortalidade nos primeiros anos de vida podem ser exacerbadas pelas condições socioeconômicas locais^(16)^. Como resultado dessas diferenças, a distribuição espacial da mortalidade neonatal não ocorre de maneira aleatória em várias regiões do mundo, e seus coeficientes têm redução mais rápida nos países desenvolvidos^(4)^. As taxas variam dentro de um mesmo território, correlacionando o papel dos espaços geográficos e suas fronteiras com as populações em risco para determinado agravo^(17)^.

O decréscimo nas taxas de mortalidade infantil brasileiras está associado ao acesso universal aos serviços de saúde oferecidos pela Estratégia de Saúde da Família, com foco nas ações de promoção e prevenção à saúde da criança^(18)^. Nesse cenário, destacam-se programas de saúde vigentes no estado, como o Mãe Coruja Pernambucana, cujo objetivo é oferecer atenção integral às gestantes usuárias do Sistema Único de Saúde (SUS) e aos seus bebês.

O comportamento da mortalidade neonatal total nas Geres V, VII, IX e XI, a partir do ano 2000, foi crescente até 2004 e, nos anos seguintes, acompanhou a tendência decrescente das demais regiões do estado até 2020. Nessas regionais, o Programa Mãe Coruja Pernambucana (PMCPE) foi implantado em 2007 (IX), 2009 (V e XI) e 2012 (VII), reafirmando a não associação da implantação deste programa com a mudança no comportamento da mortalidade nos municípios participantes.

O perfil do declínio da taxa de mortalidade neonatal em Pernambuco é semelhante ao do Brasil^(6)^. As Geres II, VI, X e XII apresentaram uma tendência decrescente e homogênea em todo o período analisado. A implantação do PMCPE nos municípios dessas regionais ocorreu em 2010, sem interferir no comportamento decrescente dessas Geres.

No estado, a implantação do Programa Mãe Coruja Pernambucana (PMCPE) em 2007 tinha como objetivo melhorar os coeficientes de mortalidade materna e infantil por meio da qualificação da assistência pré-natal, do parto e do cuidado com o recém-nascido^(14)^. Entretanto, não foram observadas mudanças ou acentuações de tendências entre as variáveis estudadas após sua implantação, um resultado semelhante ao encontrado em outro estudo realizado neste estado^(7)^.

A falta de priorização do PMCPE sobre as intervenções de maior impacto na mortalidade neonatal, juntamente com a implantação em períodos temporais diferentes em algumas Geres, pode ter contribuído para a ausência de resultados significativos sobre os coeficientes da taxa de mortalidade neonatal no período analisado^(7)^. Paralelamente, a implantação do programa de forma aleatória, sem continuidade territorial sistematizada e com foco exclusivo nas regiões com maiores coeficientes de óbito neonatal, também pode ter refletido na ausência de resultados positivos do programa sobre as variáveis relacionadas ao óbito neonatal.

Entre os constituintes da taxa de mortalidade neonatal, a velocidade de queda do componente precoce foi aproximadamente quatro vezes maior que a do tardio no estado. As Geres V, VII, IX e XI apresentaram uma tendência crescente para a mortalidade neonatal precoce entre 2000 e 2004, seguida de uma tendência decrescente desse período até o final do recorte temporal. O comportamento crescente no início da série para o componente precoce ocorre devido à ausência de serviço especializado (Geres V), ao distanciamento territorial dos polos capacitados nesse tipo de assistência (VII e XI) e a uma taxa de mortalidade elevada pré-existente já registrada em outros estudos^(6,19)^. Em Pernambuco, a mortalidade neonatal evoluiu com redução sustentada em seus componentes precoce e tardio, sendo a velocidade de queda do componente precoce superior à do total, confirmando a tendência apresentada em outros estudos^(3-7)^.

A mortalidade neonatal tardia apresentou um comportamento uniforme e decrescente em todas as Geres, exceto nas Geres V, VI e VII. Todavia, apenas a Geres V mostrou significância estatística para esse componente. O óbito neonatal tardio está associado a causas evitáveis e reduzíveis pela atenção qualificada à mulher durante a gestação, o parto e ao recém-nascido^(17)^. Na Geres V, a principal causa de internamento no público feminino em idade fértil está ligada ao adoecimento no ciclo gravídico-puerperal, confirmando os resultados obtidos nesse estudo^(20)^.

### Limitações do estudo

Este estudo apresentou como limitação a utilização de dados secundários, que dependem da completude das informações nos sistemas de informação alimentados pelos registros gerados nos serviços de saúde. Como benefícios, estudos que utilizam sistemas de informação, além de serem de baixo custo e rápidos, também possibilitam uma observação ampliada sobre determinado agravo, com a formulação de indicadores que subsidiam o planejamento e as intervenções em saúde.

### Contribuições para a área da enfermagem, saúde ou política pública

Os achados deste estudo possibilitam ampliar o conhecimento sobre o óbito neonatal em Pernambuco e evidenciam a necessidade de novas estratégias para redução do agravo, direcionadas às áreas com maior concentração de notificações. O enfermeiro, enquanto gestor em saúde, pode utilizar essas informações para efetuar o planejamento de ações de promoção em saúde direcionadas à redução da mortalidade neonatal, embasadas pela realidade situacional e, consequentemente, reduzir a distância até o cumprimento da meta 3.2 dos Objetivos de Desenvolvimento Sustentável.

## CONCLUSÕES

A tendência de mortalidade neonatal e seus componentes foram decrescentes no estado de Pernambuco. Entretanto, o componente precoce apresentou uma velocidade de redução quatro vezes maior quando comparada ao tardio. Destaca-se ainda que, apesar da redução dos coeficientes, a velocidade de redução dos óbitos neonatais não é homogênea entre todas as regiões do estado.

Em Pernambuco, mesmo após a implantação do Programa Mãe Coruja Pernambucana (PMCPE), não foram identificados resultados significativos sobre a tendência da mortalidade neonatal. O desenvolvimento de ações sistematizadas e de forma contínua desde o pré-natal até o nascimento em todo o território pernambucano pode contribuir para uma redução significativa nas taxas de mortalidade neonatal no Estado, atuando sobre as principais variáveis que interferem no óbito neonatal.

Dessa forma, foi observado que a realização de estudos com dados secundários possibilita a identificação das áreas críticas e em transição com maior necessidade de intervenção. Outro benefício desse tipo de estudo é o subsídio das informações obtidas para a construção de um planejamento estratégico direcionado à realidade local, visando à redução nos coeficientes de mortalidade neonatal.
